# Phosphatidic Acid Homeostasis and Membrane Lipid Remodeling Confer Salt Tolerance in *Zoysia japonica* by Stabilizing Metabolic Networks and a Putative SOS Signaling Activation

**DOI:** 10.3390/plants14233630

**Published:** 2025-11-28

**Authors:** Qinhao Yang, Xiangcui Zeng, Zhenzhen Liu, Zhongkuan Liu, Qiannan Hu, Mingna Li

**Affiliations:** 1 Institute of Animal Sciences, Chinese Academy of Agricultural Sciences, Beijing 100193, China; yangqinhao@cau.edu.cn (Q.Y.); xiangcui0212@163.com (X.Z.); 2 College of Grassland Science and Technology, China Agricultural University, Beijing 100193, China; liuzhenzhende@126.com; 3 Institute of Agricultural Resources and Environment Research, Hebei Academy of Agriculture and Forestry Sciences, Shijiazhuang 050051, China; zhongkuanjh@163.com

**Keywords:** *Zoysia japonica*, salt stress, non-targeted metabolomics, lipid metabolism, energy homeostasis

## Abstract

Soil salinization poses a major threat to plant growth and ecosystem sustainability. *Zoysia japonica*, a salt-tolerant turfgrass, shows promise for saline–alkali soil remediation, yet its metabolic adaptation mechanisms remain poorly understood. Here, we applied non-targeted liquid chromatography/mass spectrometry (LC/MS) metabolomics to compare the responses of salt-tolerant (accession 68) and salt-sensitive (accession 9) genotypes of *Z. japonica* under salt stress. The sensitive genotype exhibited stronger metabolic disruption, with 843 differentially accumulated metabolites (largely down-regulated), compared with 595 in the tolerant genotype (predominantly up-regulated). We identified a coordinated tolerance mechanism primarily centered on lipid remodeling and energy maintenance. The tolerant genotype enhanced membrane stability through the accumulation of saturated glycerophospholipids and an increased phosphatidylcholine/phosphatidylethanolamine (PC/PE) ratio, while maintaining phosphatidic acid (PA) homeostasis which may facilitate SOS-dependent Na^+^ efflux. It also mitigated oxidative damage by stabilizing diacylglycerol (DAG), thereby potentially limiting protein kinase C (PKC) overactivation. Furthermore, sustained cardiolipin and riboflavin metabolism supported mitochondrial energy production in the tolerant genotype. Together, these findings provide new insights into the early metabolic basis of salt tolerance in *Z. japonica*, suggesting a potential crucial role for PA-mediated regulation of SOS-dependent sodium sequestration during the initial phase of stress, and implying potential targets for breeding stress-resilient turfgrasses.

## 1. Introduction

Soil salinization has become a critical global environmental challenge, driven by both natural factors, such as climate and geology, and anthropogenic causes, including poor irrigation practices and excessive fertilizer use. In China alone, salinization affects nearly 100 million hectares, with saline–alkali soils concentrated in the North China Plain, Northeast Plain, northwestern regions, and coastal zones [1]. Salt stress impairs plant growth through osmotic and ionic stress. Elevated soil salinity increases osmotic pressure, limiting water uptake and causing physiological dehydration that suppresses leaf expansion and photosynthesis [2]. Concurrently, intracellular accumulation of Na^+^ and Cl^−^ disrupts enzymatic activity, compromises membrane integrity, disturbs ion balance, and promotes oxidative damage and metabolic dysfunction [3]. Secondary effects, including reactive oxygen species (ROS) accumulation and lipid peroxidation, further exacerbate injury [4]. Collectively, these processes restrict growth, reduce yields, and threaten both agriculture and ecosystems.

Plants counter salinity primarily through salt avoidance or tolerance. Key adaptations include the synthesis of osmoprotectants such as glycine betaine and proline to maintain turgor [5]; activation of Na^+^/H^+^ antiporters like SOS1 through the Salt Overly Sensitive (SOS) pathway to facilitate Na^+^ extrusion or vacuolar sequestration [2]; enhanced activity of ROS-scavenging enzymes including superoxide dismutase (SOD) and peroxidase (POD) [6]; and hormone-mediated regulation such as abscisic acid (ABA)-induced stomatal closure and transcriptional reprogramming [7]. These mechanisms form a multilayered defense system against salinity.

Metabolomics, especially when integrated with transcriptomics, offers a powerful means to analyze dynamic metabolic adjustments under salt stress, as demonstrated in studies on perennial ryegrass where combined metabolomic and transcriptomic profiling revealed the role of phenylpropanoid biosynthesis in root salt tolerance [8], complementing physiological studies and providing molecular evidence of tolerance mechanisms. Osmotic adjustment studies confirm the accumulation of compatible solutes such as betaine and proline in halophytes [9], with proline and betaine concentrations positively correlated with salt tolerance in alfalfa nodules [10]. Regarding membrane stability, salt stress induces phosphatidic acid (PA) accumulation and sterol changes (e.g., cholesterol), both of which affect fluidity and stability [11]. Exogenous phosphatidylcholine (PC) reduces membrane damage, lowering malondialdehyde (MDA) in peach seedlings [12], while sphingolipids like ceramides enhance rice root resistance to ionic stress [13]. Other metabolite responses include organic acids (e.g., malate and citrate), which alleviate ion toxicity via chelation or pH buffering [14]; flavonoids, particularly flavonol glycosides, which improve ROS scavenging and photoprotection [15]; and amino acid derivatives such as γ-aminobutyric acid (GABA), which contribute to osmotic adjustment, pH stability, and stress signaling [14,16]. Metabolomic evidence also highlights the balance between antioxidants (glutathione and ascorbate) and oxidative damage markers (e.g., MDA) [17,18], and shows that ABA signaling enhances adaptation by inducing osmoprotectant biosynthesis genes like proline [19]. However, the systemic remodeling of metabolic networks—particularly lipid metabolism and its integration with membrane stability, signaling, and energy supply—remains to be fully elucidated [11].

*Zoysia* species, warm-season turfgrasses with stoloniferous growth and deep root systems, are valued for high wear tolerance, stress resilience, and ecological benefits such as soil stabilization and saline–alkali land restoration [20]. *Zoysia* exhibits notable salt tolerance [21], partly due to active Na^+^ secretion through foliar salt glands that limit cellular sodium buildup [22]. Transcriptome analyses have revealed early salt stress responses in *Zoysia* roots, including genes enriched in ROS signaling, hormone regulation (ABA and auxin), and ion transport [20,23]. Under prolonged stress, pathways involving hormone signaling, photosynthesis regulation, and oxidative defense dominate [20,24]. Overexpression of the *Zoysia* vacuolar pyrophosphatase gene (*ZmVP1*) enhances salt tolerance in yeast and *Arabidopsis*, likely through vacuolar sodium sequestration, potassium balance, and antioxidant activation [25]. Conversely, salt-induced expression of *ZjPPH* accelerates chlorophyll degradation, impairing photosynthesis, while its suppression prolongs leaf greenness [26]. Chlorophyll b reductase genes *NYC1* and *NOL* are similarly upregulated under salt stress, reducing photosynthetic efficiency [27]. Transcription factors such as *ZjICE1* (bHLH class) and AP2/EREBP family members also enhance salt tolerance by upregulating antioxidant and osmoprotectant pathways [28,29]. Despite such advances, the specific contributions of metabolic pathways—including lipids, amino acids, and carbohydrates—to salt tolerance in *Zoysia* remain incompletely understood.

To address this, we systematically characterized the metabolic remodeling of *Zoysia* in response to salt stress at an early phase (12 days), a time point at which clear physiological divergence between tolerant and sensitive genotypes is already established. Based on phenotypic screening, we selected salt-tolerant accession 68 (ST68) and salt-sensitive accession 9 (SS9) [30]. We hypothesized that the tolerant genotype maintains metabolic network stability under salt stress through specific lipid remodeling. Using non-targeted liquid chromatography/mass spectrometry (LC/MS) metabolomics, we profiled leaf metabolites under control and salt treatments to (1) compare global metabolic perturbations, (2) identify key differential pathways, and (3) integrate these findings into a mechanistic model for salt tolerance. This study provides an integrated view of salt tolerance mechanisms in *Zoysia* at the metabolic level, offering new insights into stress adaptation and practical targets for breeding resilient turfgrasses.

## 2. Materials and Methods

### 2.1. Plants and Treatments

*Zoysia* grass accessions were obtained from the *Zoysia* Germplasm Resource Nursery in Jiaozhou, Shandong, China. Two genotypes with contrasting salt tolerance—ST68 and SS9, which exhibited clear differences in leaf desiccation, wilting, and growth inhibition under salt stress—were selected for this study [30]. Individual plants were transplanted into round plastic pots (25 cm diameter × 18 cm height) and grown in a controlled-environment chamber (16/8 h of light/dark; day/night 30 °C/25 °C; 60% relative humidity). During a two-month pre-culture period, plants were irrigated with Hoagland nutrient solution and trimmed regularly to ensure uniform growth. After pre-culture, uniformly sized plants were assigned to control and treatment groups (six biological replicates per group). Each biological replicate consisted of an individual pot, and leaf samples for metabolomic analysis were collected from multiple leaves within the same pot on day 12. Treatment plants were irrigated with 500 mL of NaCl solution every two days; this volume was sufficient to reach the pot’s water-holding capacity and allow for slight drainage, ensuring uniform distribution of the salt stress. The salt concentration began at 75 mM and was increased by 75 mM every second irrigation until reaching 300 mM, which was then maintained. The final concentration of 300 mM NaCl was selected based on preliminary screening that reliably induced distinct phenotypic and physiological symptoms of salt stress in *Z. japonica*. Controls received the same volume of Hoagland solution on the same schedule. Leaf samples for metabolomic analysis were collected on day 12.

### 2.2. Sample Preparation and LC-MS Analysis

Leaf material was rinsed briefly in distilled water, blotted dry, wrapped in aluminum foil, flash-frozen in liquid nitrogen, and stored at −80 °C until extraction. Eighty mg of accurately weighed frozen sample was placed into a 1.5 mL microcentrifuge tube with two stainless-steel beads. Twenty mL of each internal standard (L-2-chlorophenylalanine, 0.3 mg mL^−1^; Lyso PC17: 0, 0.01 mg·mL^−1^, both in methanol) were added, followed by extraction with 1 mL of a pre-cooled (−20 °C) mixture of methanol and water (7:3, *v*/*v*). Samples were pre-cooled at −20 °C for 2 min, homogenized at 60 Hz for 2 min, and then sonicated at room temperature for 30 min. After incubation at −20 °C for 20 min, the extracts were centrifuged at 13,000 rpm and 4 °C for 10 min. A 300 μL aliquot of the supernatant was transferred and dried under a stream of nitrogen. The residue was reconstituted in 200 μL of methanol and water (1:4, *v*/*v*), vortexed for 30 s, and sonicated for 2 min. After a final centrifugation (13,000 rpm, 4 °C, 10 min), a 150 μL aliquot of the supernatant was filtered through a 0.22 μm membrane into an LC vial and stored at −80 °C. Quality-control (QC) samples were prepared by pooling equal aliquots from all extracts.

Chromatographic separation was performed on an ACQUITY UPLC system (Waters Corporation, Milford, CT, USA) coupled to an AB SCIEX Triple TOF 5600 mass spectrometer (AB SCIEX, Framingham, MA, USA). Separation was achieved using an ACQUITY UPLC BEH C18 column (1.7 μm, 2.1 × 100 mm) maintained at 45 °C. The mobile phase consisted of (A) water with 0.1% formic acid and (B) a mixture of acetonitrile and methanol (2:3, *v*/*v*) containing 0.1% formic acid. The gradient program was: 0.0 min, 5% B; 2.0 min, 20% B; 4.0 min, 25% B; 9.0 min, 60% B; 17.0 min, 100% B; 19.0 min, 100% B; 19.1 min, 5% B; 20.1 min, 5% B. The flow rate was 0.4 mL·min^−1^, column temperature 45 °C, injection volume 5 μL, and sample tray 4 °C.

### 2.3. MS Data Acquisition and Processing

A total of 29 samples were analyzed by mass spectrometry, comprising 24 experimental samples (2 genotypes × 2 treatments × 6 biological replicates) and 5 QC samples pooled from all extracts. Mass spectra were acquired in both ESI positive and negative ion modes over a mass scan range of *m*/*z* 70–1000. The mass spectrometer parameters were set as follows: nebulizer gas (GS1), 40 psi; auxiliary gas (GS2), 40 psi; curtain gas, 35 psi; ion source temperature, 550 °C; ion spray voltage, 5500 V (positive) and 4500 V (negative); declustering potential, 100 V (positive) and −100 V (negative). For full MS scans, the collision energy was set to 10 eV (positive) and −10 eV (negative). For product ion scans, the mass range was *m*/*z* 50–1000 with a collision energy of 30 eV. QC samples were injected every ten runs to monitor system performance. Raw data were acquired using UNIFI software (Waters Corporation, version 1.8.1) and processed using Progenesis QI (Waters Corporation, version 2.3) with precursor tolerance set to 5 ppm, product tolerance set to 10 ppm, product ion threshold set to 5%, and retention time tolerance set to 0.02 min. Isotopic peaks were excluded; noise level was set to 10, and the minimum intensity threshold to 15% of the base peak. A three-dimensional data matrix (*m*/*z*, retention time, normalized peak intensity) was generated; ions with zero intensity in >60% of samples were removed. Metabolite identification was conducted by querying accurate mass (mass error < 5 ppm) and MS/MS fragmentation patterns against the Human Metabolome Database (HMDB), LipidMaps (v2.3), and METLIN databases. Putative identifications with a confidence score of less than 30 (out of 60) were discarded. Finally, to ensure data reproducibility, ions with a relative standard deviation (RSD) > 0.4 in the QC samples were removed.

### 2.4. Statistical Analysis and Metabolite Identification

Peak lists from positive and negative ion modes were merged and imported into SIMCA 14.0 for multivariate analysis. Data were mean-centered and Pareto-scaled before principal component analysis (PCA) and orthogonal partial least squares discriminant analysis (OPLS-DA). A seven-round cross-validation was applied to reduce overfitting. Metabolites with variable importance in projection (VIP) > 1 and Student’s *t*-test *p*-value < 0.05 were considered significant. A consolidated matrix of retention time, *m*/*z*, and normalized peak area was compiled for downstream statistical and pathway analyses.

## 3. Results

### 3.1. Metabolomic Profiling Reveals Greater Global Perturbation in the Sensitive Genotype

**Figure 1:**
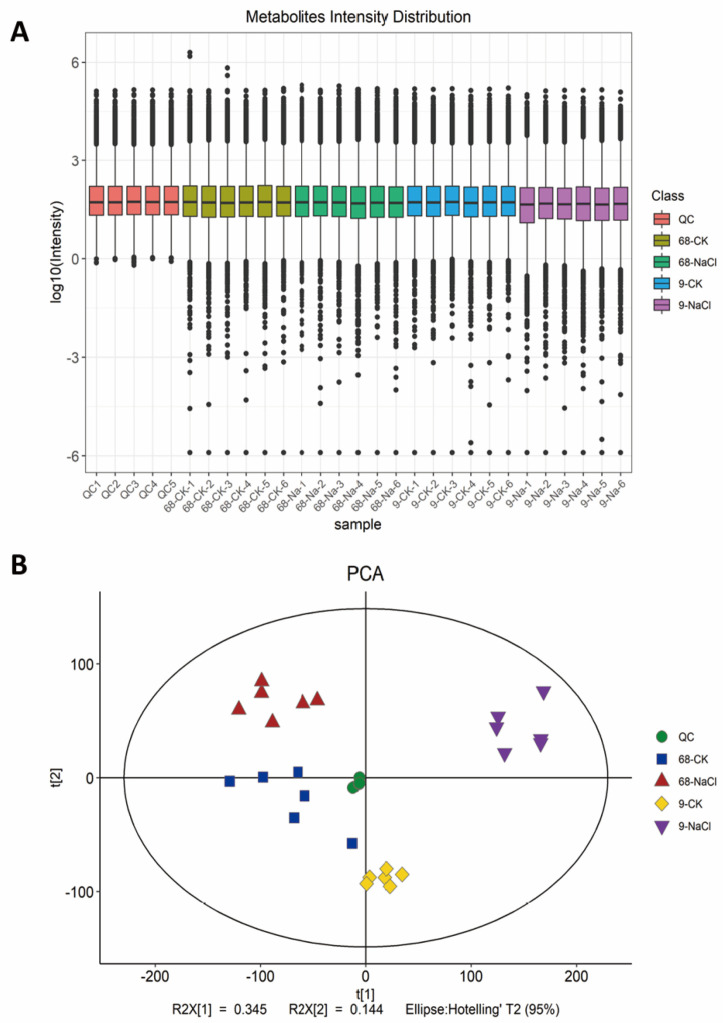
Metabolic profiling of salt-tolerant (ST68) and salt-sensitive (SS9) *Z. japonica* under 300 mM NaCl stress. (**A**) Distribution of metabolite intensities. Quality control (QC) samples are tightly clustered, demonstrating high reproducibility. ST68 showed similar intensity distributions between control and salt-treated groups, whereas SS9 displayed significant decreases and greater variability under salt stress. (**B**) Principal component analysis (PCA) score plot. QC samples clustered near the origin. Clear separation between control and salt-treated groups was observed in SS9 but not in ST68.

Our previous finding [30] revealed dramatic differences between SS9 and ST68 plants under high salinity: SS9 plants displayed severe leaf tip burning, wilting, and extensive leaf drying, ultimately leading to plant death, whereas ST68 plants maintained a relatively healthy appearance with only slight leaf dehydration. To dissect physiological responses to salt stress in ST68 and SS9, we applied non-targeted LC/MS metabolomics to leaves from NaCl-treated and control (CK) plants. Data preprocessing and quality control produced a tight clustering of QC samples (Figure 1A), confirming analytical stability. The metabolite intensity distribution (Figure 1A) showed near-identical boxplots for CK and NaCl-treated samples of ST68, whereas the NaCl-treated group of SS9 exhibited a markedly lower median and a wider spread, indicating greater global metabolic disruption. PCA corroborated these observations: QC samples clustered near the origin within the 95% confidence ellipse, ST68’s CK and NaCl groups largely overlapped along PC1 with only minor separation on PC2, while SS9’s CK and NaCl groups were clearly separated along both PC1 and PC2 (Figure 1B). Together, these results indicate that salt stress provokes substantially greater perturbation of the metabolic network in SS9 than in ST68.

### 3.2. Predominant Metabolite Up-Regulation Underpins Metabolic Stability in Tolerant Genotype

**Figure 2:**
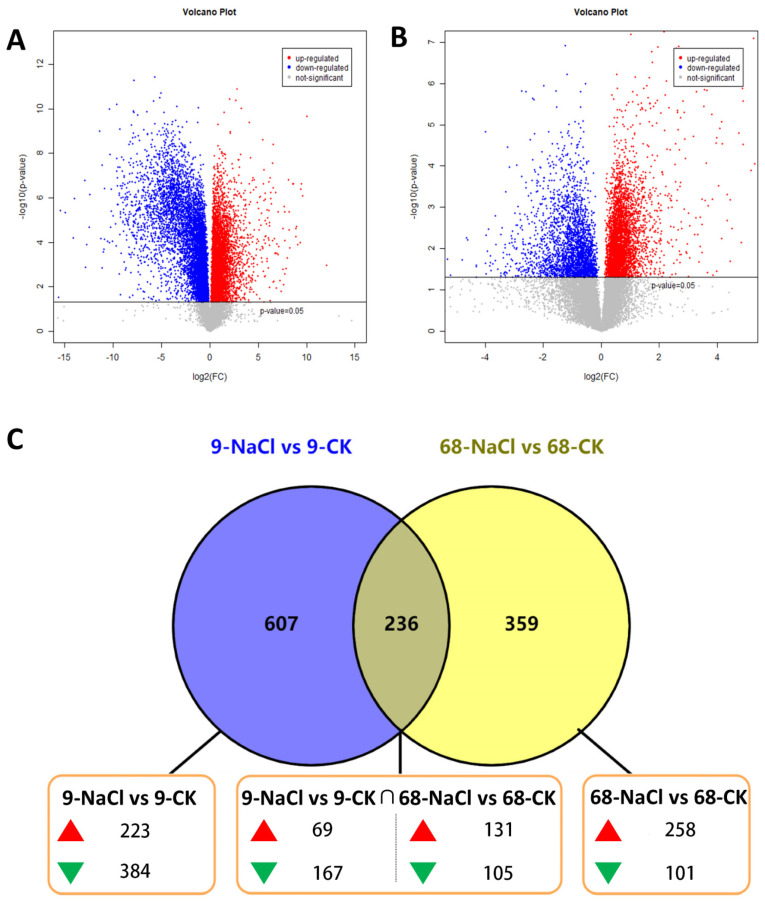
Number and variation characteristics of differential metabolites. (**A**) Volcano plot of differential metabolites in SS9 under 300 mM NaCl stress. (**B**) Volcano plot of differential metabolites in ST68. (**C**) Venn diagram of differential metabolites. SS9 had 607 unique differential metabolites (223 up, 384 down), ST68 had 359 (258 up, 101 down), and 236 were shared. Among shared metabolites, most were upregulated in ST68 but downregulated in SS9.

Univariate analysis revealed marked differences in both the number and direction of differential metabolites between SS9 and ST68 (Figure 2A,B). Venn analysis (Figure 2C) showed distinct distributions: SS9 harbored 607 unique differential metabolites (223 up-regulated and 384 down-regulated), ST68 had 359 unique metabolites (258 up-regulated and 101 down-regulated), and the two lines shared 236 differential metabolites. Among shared metabolites, ST68 showed a predominance of up-regulation (131 up vs. 105 down), whereas SS9 exhibited a predominance of down-regulation (69 up vs. 167 down). These patterns are consistent with the idea that ST68 preserves a more stable metabolic state under salt stress, while SS9 mounts a broader down-regulatory response.

### 3.3. Cluster Analysis Identifies Lipid Remodeling as a Central Response, with PA Playing a Pivotal Role

**Figure 3:**
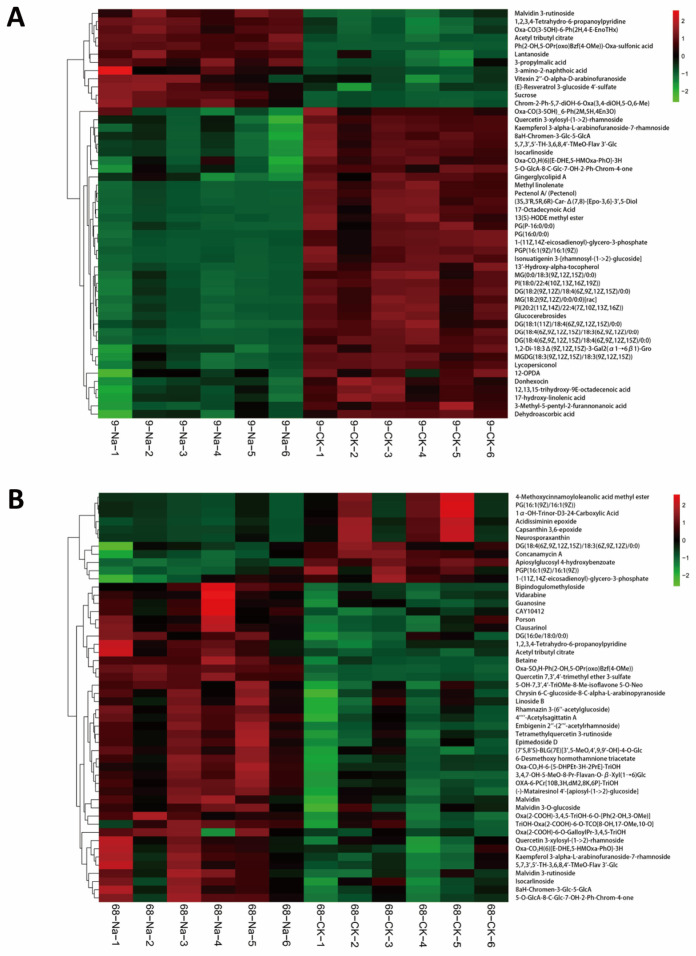
Cluster analysis of the top 50 differential metabolites in *Z. japonica* under 300 mM NaCl stress. (**A**) Heatmap of SS9. Strong divergence was observed between the NaCl-treated and control groups. (**B**) Heatmap of ST68. Expression changes were more moderate under salt stress compared to SS9.

We screened differential metabolites using a combined multivariate and univariate strategy (OPLS-DA VIP > 1 and *t*-test *p* < 0.05), then performed hierarchical clustering and plotted a heatmap of the top 50 metabolites ranked by VIP (Figure 3A,B). The heatmap reveals pronounced separation between NaCl and CK in SS9: flavonoids and heterocyclic compounds were largely up-regulated under NaCl, whereas lipids were strongly down-regulated. By contrast, metabolic shifts between NaCl and CK in ST68 were more moderate (Figure 3B), though decreases in membrane lipid components and increases in flavonoids and glycosylated secondary metabolites were still evident. Category analysis showed that lipids and lipid-like molecules constituted the largest class of differential metabolites. In ST68, 246 differential lipid species were identified, of which only 23 were down-regulated. Notably, among the total of 36 PAs species detected in ST68, only six were significantly down-regulated. The fact that the overwhelming majority of PA species (30 out of 36) were not significantly down-regulated provides strong evidence for the maintenance of PA homeostasis under salt stress. In SS9, 117 of 140 differential lipid species (including 23 PA species) were significantly down-regulated, with mean decreases of ~3–6-fold. These results suggest that lipids—particularly PA—may act as central players in the salt-stress response of *Z. japonica*.

### 3.4. Correlation Analysis Uncovers a More Coordinated Metabolic Response in the Sensitive Genotype

**Figure 4:**
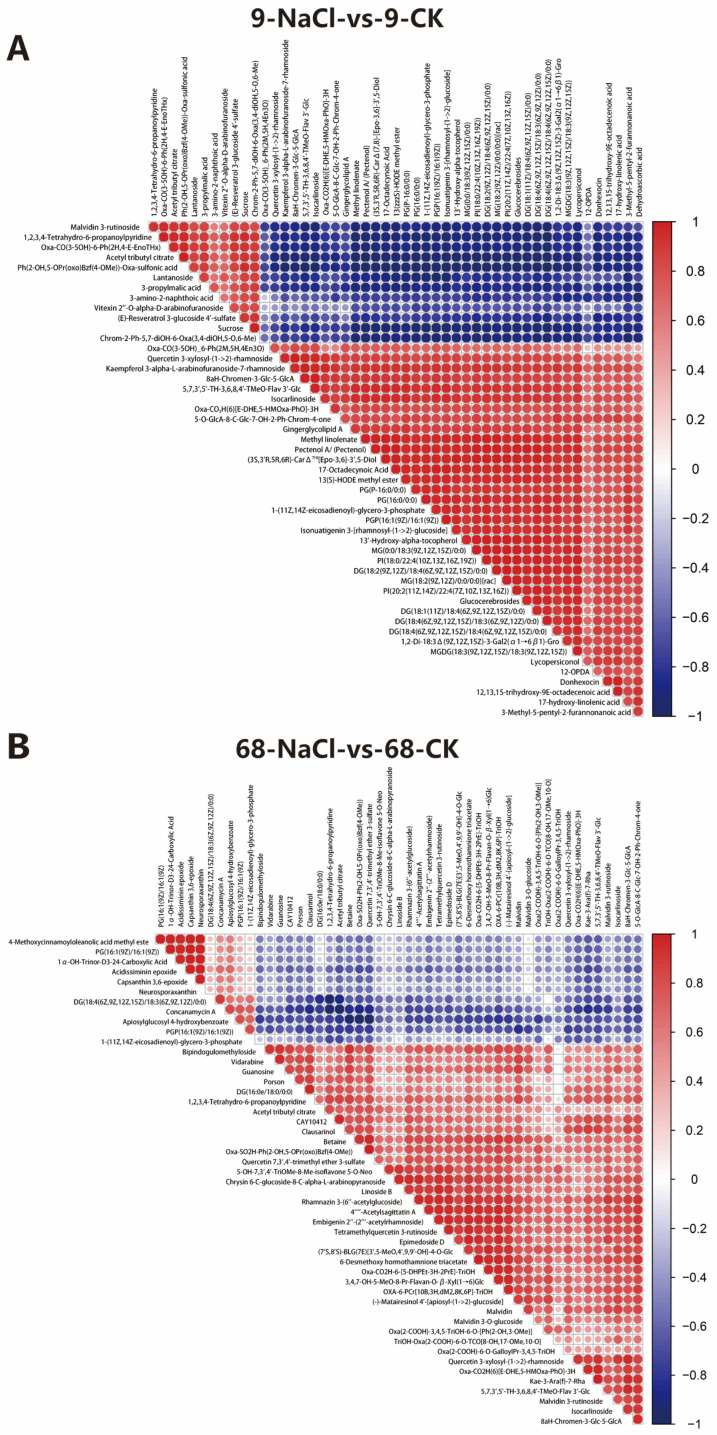
Correlation analysis of differential metabolites in *Z. japonica* under 300 mM NaCl stress. (**A**) Correlation heatmap of top differential metabolites in SS9. (**B**) Correlation heatmap of top differential metabolites in ST68. Correlation coefficients among metabolites were generally higher in SS9 than in ST68.

To explore coordinated changes within the metabolic network, Pearson correlation analysis was performed on the top 50 VIP metabolites and visualized as correlation heatmaps (Figure 4A,B). Correlation coefficients among differential metabolites were generally higher in SS9 (Figure 4A) than in ST68 (Figure 4B), indicating that salt stress induces stronger, more concerted shifts in the metabolome of the sensitive line, consistent with the greater overall perturbation observed above.

### 3.5. Tolerant Genotype Couples Lipid with Energy Metabolism to Sustain Salt Stress Response

**Figure 5:**
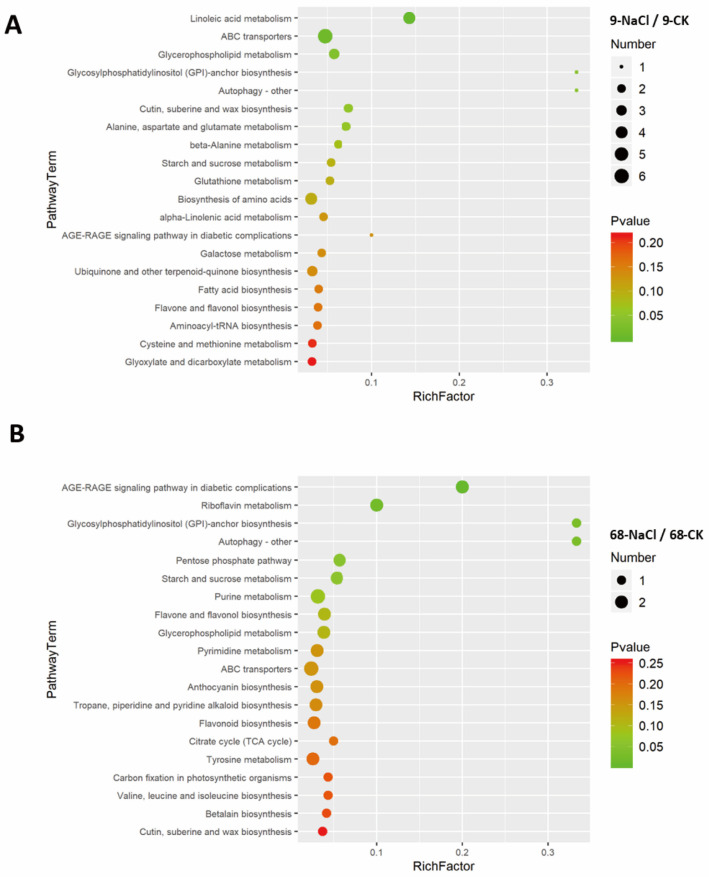
Enriched metabolic pathways of differential metabolites in *Z. japonica* under 300 mM NaCl stress. (**A**) Bubble chart of enriched KEGG pathways in SS9. (**B**) Bubble chart of enriched KEGG pathways in ST68. Lipid metabolism pathways were the most enriched in both genotypes. SS9 responded primarily through lipid and amino acid metabolism, whereas ST68 exhibited an integrated energy–lipid response.

KEGG pathway enrichment—conducted using *Oryza sativa japonica* pathways as the background—identified 117 differential metabolites significantly enriched across 54 pathways in ST68 (*p* < 0.05) and 137 metabolites enriched across 59 pathways in SS9. Bubble plots summarize the most significant pathways (Figure 5A,B). In SS9 (Figure 5A), lipid-related pathways dominated the enrichment landscape: seven lipid pathways (including linoleic acid metabolism, α-linolenic acid metabolism, glycerophospholipid metabolism, and GPI-anchor biosynthesis) comprised ~35% of enriched terms; amino acid pathways (alanine, aspartate, and glutamate metabolism; β-alanine metabolism) were also enriched. In ST68 (Figure 5B), four lipid pathways (glycerophospholipid metabolism, GPI-anchor biosynthesis, and cutin/suberin/wax biosynthesis) accounted for ~20% of enriched terms. In contrast, riboflavin metabolism and the pentose phosphate pathway were notably enriched, implicating energy metabolism. Collectively, these results suggest that SS9 responds primarily via lipid and amino-acid metabolic remodeling, whereas ST68 engages a coordinated energy–lipid adaptive program, reflecting distinct strategies of membrane lipid remodeling under salt stress.

### 3.6. Proposed Model of a PA-Mediated Metabolic Network for Salt Tolerance

**Figure 6:**
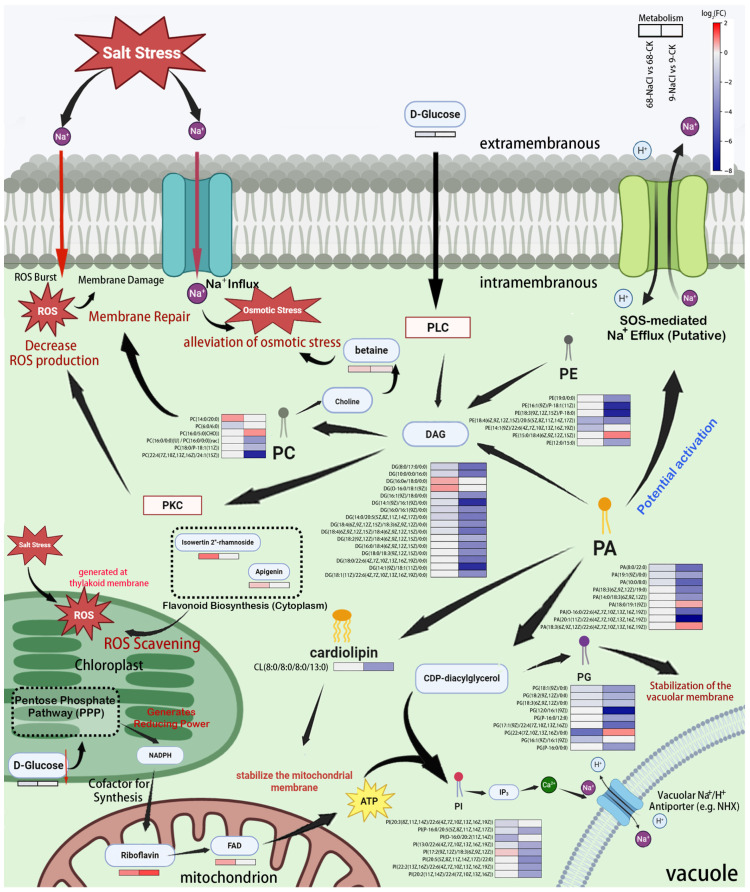
PA-centered lipid metabolic network in *Z. japonica* leaves under 300 mM NaCl stress. Metabolites are color-coded by accumulation pattern: red for upregulation, blue for downregulation, and gray for no significant change, with color intensity reflecting fold change magnitude. This network highlights the distinct remodeling of glycerophospholipid metabolism between ST68 and SS9, underscoring its role in regulating membrane integrity, signaling, and energy homeostasis.

To integrate the observed metabolic shifts, we developed a conceptual model of the salt-stressed plant cell (Figure 6). The schematic highlights major pathways and their interconnections, centering on the dual roles of PA) and glycerophospholipid metabolism. Na^+^ influx is depicted as triggering membrane damage and ROS production. The model emphasizes the conversion of phosphatidylcholine (PE) to phosphatidylethanolamine (PC) via diacylglycerol (DAG), which elevates the PC/PE ratio to stabilize membranes. From PC-derived choline, betaine synthesis contributes to osmotic adjustment. The D-glucose downregulation may reflect its redirection into the chloroplastic pentose phosphate pathway (PPP), potentially generating NADPH and ribose-5-phosphate to support riboflavin and (FAD) biosynthesis–a proposed link requiring further experimental validation. D-Glucose is also shown to initiate a signaling cascade involving phospholipase C (PLC), DAG, and protein kinase C (PKC), which may regulate ROS dynamics. Furthermore, the model incorporates the observed upregulation of flavonoids, such as apigenin and its derivatives, proposing their role in scavenging ROS generated from multiple sites. PA is proposed to activate SOS-mediated Na^+^ efflux. It also serves as a precursor for cardiolipin (CL) and phosphatidylglycerol (PG), reinforcing membrane integrity, and as a substrate for phosphatidylinositol (PI), which drives inositol trisphosphate (IP3)––Ca^2+^ signaling and vacuolar Na^+^ sequestration via vacuolar Na^+^/H^+^ antiporters (e.g., NHX). Metabolites are color-coded to reflect their accumulation patterns in the salt-tolerant genotype under stress.

## 4. Discussion

This study compared the early metabolic response patterns of salt-tolerant and salt-sensitive *Z. japonica* varieties under a short-term (12 days) salt stress that induced clear phenotypic and physiological symptoms of stress, revealing striking differences in membrane structural maintenance, signal transduction, and network stability. These distinctions provide new insights into the molecular basis of salt adaptation. In particular, the contrasting accumulation of core lipids and osmolytes between genotypes (Table 1) underscores the central role of glycerophospholipid remodeling, with PA functioning as a key regulatory hub.

### 4.1. Remodeling of Glycerophospholipid Metabolic Network Centered on Phosphatidic Acid

ST68 may enhance membrane stability by accumulating medium- and long-chain saturated glycerophospholipids (C14–C20). For example, PC (14:0/20:0) was significantly upregulated (log_2_FC = 0.721), which could increase bilayer order through stronger van der Waals interactions within the hydrophobic core [31], potentially reducing ion leakage and protecting against ROS-induced lipid peroxidation. Such strategies are consistent with those of halophytes, which preserve membrane integrity by increasing saturated fatty acyl chains (e.g., C14:0, C20:0) [32]. Similarly, woody plants enhance polyprenol biosynthesis under salt stress, improving membrane fluidity and lowering ion permeability [33]. By contrast, SS9 suffered degradation of polyunsaturated very-long-chain lipids (≥C20 with ≥4 double bonds), such as PC (22:4/24:1) (log_2_FC = −6.700) and PA (20:1/22:6) (log_2_FC = −8.580). Their loss could have impaired fluidity control, and the resulting peroxidation products (e.g., malondialdehyde) may disrupt plasma membrane H^+^-ATPase activity, potentially intensifying ion leakage and initiating a feedback loop of oxidative damage [34].

Enrichment analysis of glycerophospholipid metabolism revealed that PC production primarily arises from PE or PA conversion (Figure 6). In ST68, PC levels increased while PE decreased (e.g., PE (18:4/20:5), log_2_FC = −2.213; PE (14:1/22:6), log_2_FC = −1.788). The resulting increase in the PC/PE ratio likely reduced membrane curvature stress, thereby stabilizing bilayer organization [35] and minimizing ROS-induced structural injury. This mechanism appears conserved: drought-preconditioned tall fescue also enhanced membrane stability under heat stress by upregulating PC (e.g., PC (36:4)) and downregulating PE [36]. In ST68, this remodeling was coupled with maintenance of the PA pool (e.g., PA (8:0/22:0), log_2_FC = −0.799; PA (19:1/0:0), log_2_FC = −0.640; PA (10:0/8:0), log_2_FC = −0.595). Saturated PA species may stabilize SOS2 kinase, which could promote SOS1-mediated Na^+^ efflux [37]. Thus, the PA homeostasis observed in ST68 could potentially contribute to the activation of the SOS pathway, a key mechanism for Na^+^ extrusion in plants. This is consistent with the established model that the SOS pathway, mediating Na^+^ extrusion, represents a cornerstone of salt tolerance in plants, including turfgrasses [38]. In contrast, SS9 showed a reverse trend, with PC broadly downregulated and PE elevated (e.g., PE (15:0/18:4), log_2_FC = 0.936). This, coupled with severe PA depletion (e.g., PA (8:0/22:0), log_2_FC = −4.448; PA (19:1/0:0), log_2_FC = −2.743; PA (10:0/8:0), log_2_FC = −4.702) and DAG downregulation (e.g., DG (8:0/17:0/0:0), log_2_FC = −4.354; DG (10:0/0:0/16:0), log_2_FC = −4.202), could disrupt PC resynthesis [39] and ultimately inactivated SOS signaling.

As shown in the PA-centered network (Figure 6), PA also serves as a precursor for PG, PI, and CL. Its depletion, therefore, disrupts multiple downstream functions [40]. PA can be converted through CDP-diacylglycerol to PGP and then PG. ST68 maintained expression of saturated PG species (e.g., PG (P-16:0/12:0), PG (P-16:0/0:0)), whose ether bonds may scavenge ROS and protect tonoplast integrity [41], facilitating Na^+^ sequestration. CDP-diacylglycerol also gives rise to PI and CL. PI hydrolysis produces IP_3_, which activates vacuolar Ca^2+^ channels to regulate NHX-mediated Na^+^ compartmentalization [2]. CL, localized to the mitochondrial inner membrane, stabilizes energy metabolism [40].

The remodeling of glycerophospholipid metabolism also had downstream effects on other metabolic pools. The increase in PC levels in ST68 not only contributed to membrane stability but also provided abundant precursor (choline) for the observed accumulation of betaine (Table 1). Conversely, the severe degradation of PC and disruption of phospholipid metabolism in SS9 likely limited choline availability, leading to a fundamental strategic divergence in osmotic adjustment. In ST68, membrane integrity is prioritized through lipid remodeling, with betaine synthesis directly coupled to stable phosphatidylcholine metabolism. This coordinated strategy simultaneously ensures osmotic balance and membrane fortification. In stark contrast, SS9, experiencing a collapse in membrane lipid homeostasis, resorts to a massive accumulation of proline. While proline is a versatile protectant known to contribute to osmotic adjustment, redox balance, and protein stabilization [42,43,44], its extreme accumulation in SS9 likely signals a desperate, last-resort mechanism during catastrophic cellular dehydration [45]. This demonstrates that effective osmotic adjustment depends on core lipid metabolic stability—a key facet of ST68’s superior salt tolerance. However, the direct physiological contribution of these osmoprotectant changes to osmotic adjustment remains to be functionally validated.

### 4.2. DAG-PKC Signaling Axis in Oxidative Stress Regulation

In ST68, KEGG enrichment analysis revealed the “AGE-RAGE signaling pathway in diabetic complications”. Although defined in mammalian pathological context, its core downstream signaling components—PLC, DAG, and PKC—represent a highly conserved signaling module across kingdoms. Our metabolomic data, which showed distinct dynamics of D-glucose and the DAG pool between the genotypes, allow us to hypothesize that a functionally analogous PLC-DAG-PKC signaling axis may be involved in regulating the oxidative stress response during salt stress in plants. In this hypothetical model, salt stress may lead to the activation of PLC, which hydrolyzes phosphatidylinositol 4,5-bisphosphate to generate DAG IP_3_. While IP_3_ can trigger intracellular Ca^2+^ release, our data show a marked difference in DAG dynamics between the genotypes. In ST68, D-glucose was downregulated (log_2_FC = −0.514), and DAG content declined slightly (~1-fold across the 8 detected components). By contrast, SS9 exhibited sharp DAG depletion, with 22 components reduced 3–6 fold. Given that DAG is a key activator of PKC, these results could be interpreted to suggest that the PLC–DAG–PKC axis, rather than IP_3_–Ca^2+^ signaling, might represent a major route of ROS generation under salt stress. ST68 may suppress excessive PKC activation, thereby potentially limiting ROS accumulation, whereas DAG collapse in SS9 could amplify ROS production [46]. The conserved nature of PLC-PKC signaling in plants, such as its documented role in regulating NADPH oxidases (e.g., *AtrbohD/F* in *Arabidopsis*), lends credence to the potential functionality of this axis in salt stress responses [47].

Furthermore, the upregulation of flavonoids in ST68, including apigenin (log_2_FC = 0.358) and Isowertin 2′′-rhamnoside (log_2_FC = 1.020), indicates enhanced direct ROS scavenging capacity, consistent with findings in *Atriplex canescens* where flavonoids are involved in salt tolerance through ROS scavenging [48] and in rice where hydrogen sulfide enhances the flavonoid early warning system to cope with stress [49]. This response likely works in concert with the proposed DAG-PKC regulation, forming a multi-layered defense against oxidative damage from both signaling and impaired photosynthesis.

### 4.3. Cardiolipin and Riboflavin Metabolism Maintain Mitochondrial Energy Supply

KEGG enrichment revealed a significant enhancement of the PPP in ST68, alongside a marked decrease in D-glucose, suggesting a redirection of carbon into the chloroplastic PPP to produce NADPH and pentose precursors, a response consistent with observations in other salt-stressed plants [50]. The NADPH generated is essential for chloroplastic redox homeostasis, while the parallel generation of ribose-5-phosphate provides a critical precursor for the biosynthesis of riboflavin and FAD [51]. The concurrent enhancement of the PPP and upregulation of riboflavin (log_2_FC = 0.894) and FAD (log_2_FC = 0.609) suggest a potential metabolic link wherein chloroplastic PPP activity supports the synthesis of these essential mitochondrial cofactors. This presents a compelling hypothesis for future validation.

Cardiolipin (CL), known in microbes to enhance membrane thermal stability and reduce ion permeability, is a critical component of the mitochondrial inner membrane. In ST68, riboflavin metabolism was significantly enriched, and CL levels remained stable, likely sustaining mitochondrial complexes and preserving electron transport efficiency. Riboflavin was upregulated, promoting FAD synthesis, which could enhance ATP production efficiency [52] and thus stabilize energy metabolism under salt stress. By contrast, SS9 exhibited strong CL downregulation (e.g., CL (8:0/8:0/8:0/13:0), log_2_FC = −2.958), which may compromise mitochondrial membrane integrity and reduce energy output [53].

The proposed model (Figure 6) integrates these observations into a hypothetical framework, highlighting the central role of PA lipid remodeling in coordinating membrane stability, signaling, and energy metabolism under salt stress. This model serves primarily to generate future hypotheses, such as testing the direct impact of PA on SOS kinase activity or manipulating key lipid pathways to validate their functional roles in enhancing salt tolerance in *Zoysia*.

## 5. Conclusions

This non-targeted metabolomics study of the early salt stress response provides evidence that salt tolerance in *Z. japonica* is associated with the coordinated regulation of lipid metabolism and mitochondrial energy balance. The salt-sensitive genotype SS9 exhibited broader metabolic disruption (843 differential metabolites) than the tolerant ST68 (595 metabolites), indicating more severe network destabilization under salt stress. ST68 appears to strengthen membrane integrity by accumulating saturated glycerophospholipids and increasing the PC/PE ratio, while maintaining PA homeostasis, potentially facilitating SOS-mediated Na^+^ efflux. It also limited oxidative damage by preserving DAG pools, which could potentially suppress PKC overactivation. Moreover, stable CL and riboflavin metabolism in ST68 are consistent with supported mitochondrial energy production, contrasting with the impaired energy metabolism observed in SS9. Although the present study focused on the early phase of salt stress, it did not assess recovery responses. The observed metabolic stability (e.g., PA homeostasis, elevated PC/PE ratio, and sustained energy metabolism) provides crucial support for cellular function and underscores its role as an active acclimation response rather than a passive path to cell death. This early metabolic reprogramming potentially lays the groundwork for subsequent long-term morphological and transcriptional adaptation. Together, these findings point to key metabolic nodes—such as PA homeostasis, PC/PE remodeling, and the DAG–PKC signaling axis—that may underpin a mechanistic foundation and potential targets for breeding salt-tolerant turfgrasses suited to saline–alkali land restoration. Future studies integrating recovery experiments with dynamic multi-omics analyses will be valuable to validate the reversibility and long-term contribution of these metabolic changes.

## Figures and Tables

**Table 1:** Key metabolites involved in salt stress responses in contrasting *Z. japonica* genotypes.

Metabolite Pathway	Metabolite	Log_2_(FC)	
		SS9(NaCl/CK)	ST68(NaCl/CK)
Membrane Lipid Remodeling and Stability	PC (14:0/20:0)	–	0.721
	PC (22:4/24:1)	−6.700	–
	PE (18:4/20:5)	−3.430	−2.213
	PE (14:1/22:6)	–	−1.788
	PE (15:0/18:4)	0.936	–
	CL (8:0/8:0/8:0/13:0)	−2.958	–
Lipid Signaling Molecules	PA (8:0/22:0)	−4.448	−0.799
	PA (19:1/0:0)	−2.743	−0.640
	PA (10:0/8:0)	−4.702	−0.595
	PA (20:1/22:6)	−8.580	–
	DG (8:0/17:0/0:0)	−4.354	−1.089
	DG (10:0/0:0/16:0)	−4.202	−0.908
Antioxidant Metabolites	Apigenin	–	0.358
	Isowertin 2′′-rhamnoside	–	1.020
Osmoprotectants	Betaine	0.159	0.277
	L-Proline	3.688	–
Energy and Cofactor Metabolism	Riboflavin	1.402	0.894
	FAD	–	0.609
	D-Glucose	–	−0.514

SS9 and ST68 represent salt-sensitive and salt-tolerant accessions, respectively. NaCl/CK indicate comparisons between salt-treated and control groups. Values represent log_2_(fold change). A dash (–) indicates that the metabolite was not detected or did not meet significance criteria. Abbreviations: PC, phosphatidylcholine; PE, phosphatidylethanolamine; PA, phosphatidic acid; DG, diacylglycerol; CL, cardiolipin; FAD, flavin adenine dinucleotide.

## Data Availability

The original contributions presented in this study are included in the article. Further inquiries can be directed to the corresponding author(s).
